# Systematical Identification of Breast Cancer-Related Circular RNA Modules for Deciphering circRNA Functions Based on the Non-Negative Matrix Factorization Algorithm

**DOI:** 10.3390/ijms20040919

**Published:** 2019-02-20

**Authors:** Shuyuan Wang, Peng Xia, Li Zhang, Lei Yu, Hui Liu, Qianqian Meng, Siyao Liu, Jie Li, Qian Song, Jie Wu, Weida Wang, Lei Yang, Yun Xiao, Chaohan Xu

**Affiliations:** 1 College of Bioinformatics Science and Technology, Harbin Medical University, Harbin 150081, China; bioccwsy@gmail.com (S.W.); xiapeng231515@outlook.com (P.X.); Bio_MiniZhang@outlook.com (L.Z.); biomathnmghebyulei@outlook.com (L.Y.); liuhui870320@gmail.com (H.L.); mqq1992hmu@outlook.com (Q.M.); liusiyao29@outlook.com (S.L.); jacklee2THU@outlook.com (J.L.); songqian@hrbmu.edu.cn (Q.S.); wujie_bio@outlook.com (J.W.); zjhzwang@outlook.com (W.W.); 2 Key Laboratory of Cardiovascular Medicine Research, Ministry of Education, College of Pharmacy, Harbin Medical University, Harbin 150081, China

**Keywords:** Circular RNA (circRNA), breast cancer, non-negative matrix factorization (NMF) algorithm

## Abstract

Circular RNA (circRNA), a kind of special endogenous RNA, has been shown to be implicated in crucial biological processes of multiple cancers as a gene regulator. However, the functional roles of circRNAs in breast cancer (BC) remain to be poorly explored, and relatively incomplete knowledge of circRNAs handles the identification and prediction of BC-related circRNAs. Towards this end, we developed a systematic approach to identify circRNA modules in the BC context through integrating circRNA, mRNA, miRNA, and pathway data based on a non-negative matrix factorization (NMF) algorithm. Thirteen circRNA modules were uncovered by our approach, containing 4164 nodes (80 circRNAs, 2703 genes, 63 miRNAs and 1318 pathways) and 67,959 edges in total. GO (Gene Ontology) function screening identified nine circRNA functional modules with 44 circRNAs. Within them, 31 circRNAs in eight modules having direct relationships with known BC-related genes, miRNAs or disease-related pathways were selected as BC candidate circRNAs. Functional enrichment results showed that they were closely related with BC-associated pathways, such as ‘KEGG (Kyoto Encyclopedia of Genes and Genomes) PATHWAYS IN CANCER’, ‘REACTOME IMMUNE SYSTEM’ and ‘KEGG MAPK SIGNALING PATHWAY’, ‘KEGG P53 SIGNALING PATHWAY’ or ‘KEGG WNT SIGNALING PATHWAY’, and could sever as potential circRNA biomarkers in BC. Comparison results showed that our approach could identify more BC-related functional circRNA modules in performance. In summary, we proposed a novel systematic approach dependent on the known disease information of mRNA, miRNA and pathway to identify BC-related circRNA modules, which could help identify BC-related circRNAs and benefits treatment and prognosis for BC patients.

## 1. Introduction

Breast cancer (BC) is the most frequent malignancy in women, affecting more than 10% of women in western countries [1]. To improve the BC diagnosis and therapy with efficiency, it is imperative to explore the molecular mechanisms of BC pathogenesis [2,3]. Therefore, some biological biomarkers involved in the development of BC, including mRNAs, lncRNAs and miRNAs, have been detected [4,5,6]. Further, several studies have shown that their corresponding molecular modules play important roles in BC [7,8,9]. How to efficiently identify these molecular modules that could be potentially used as diagnostic markers and therapeutic targets has been a big challenge.

With the enormous development in the field of high-throughput RNA sequencing technology, a novel class of endogenous RNA, circular RNA (circRNA), has been extensively studied [10,11]. Some researches demonstrated that circRNAs could be involved in many biological processes, including regulation of transcription [12], neuronal development [13], cell cycle control [14], and tumorigenesis [15,16]. For instance, Simon J. Conn et al. suggested that circRNAs biogenesis could be modified during the human epithelial-mesenchymal transition (EMT), and more than 30% productions of circRNAs were dynamically controlled by the alternative splicing [17]. In addition, Guarnerio J. et al. discovered that the generation of fusion circRNAs from chromosomal translocations displayed remarkable ability in promoting cellular transformation in vitro and initiate tumors [15]. Liang et al. found that circRNA, hsa_circ_0008717 (namely circ-ABCB10), was significantly upregulated in BC tissue, and knockdown of this circRNA could restrain the proliferation of BC cells [18]. Fang et al. disclosed that the delivery of a circRNA circ-Ccnb1 suppressed the effect of p53 mutations and enhance tumor progression in BC patients [19]. The identification of circRNA biomarkers largely benefited the in-depth exploration and investigation of the developmental mechanisms of BC and provided more promises for BC patients’ diagnosis and therapy [20,21,22,23].

Although some advance in biological protocols has been made, it is time-consuming and expensive to identify BC-circRNAs only by using experimental technologies. Thus, some systematical approaches have been developed and proposed to identify BC-related circRNAs. Lu et al. identified 1155 differentially expressed circRNAs in BC tissues through analysis of a genome-wide circRNA profile data and found the expression levels of six circRNAs were related to BC which participated in cancer-related pathways [24]. Chen et al. identified the functional roles of circEPSTI1 on proliferation, clonal formation, and apoptosis in three triple-negative breast cancer (TNBC) cell lines by knocking down experiments. They confirmed that circEPSTI1 binds to miR-4753 and miR-6809 as a miRNA sponge to affect TNBC proliferation and apoptosis [25]. Many BC-related circRNAs had been identified and promoted the development of circRNA research. However, the disease circRNA list of BC is relatively incomplete and the systematical researches for their relevant functions remain poor, which handles the BC diagnosis and therapy.

Thus, we integrated circRNA-mRNA, miRNA-mRNA, and pathway-mRNA data to identify BC-related circRNA modules based on a non-negative matrix factorization (NMF) algorithm [26], and deciphered relevant circRNA functions. NMF has been demonstrated to be a powerful tool for detecting modules in heterogeneous multi-omics data, and biological entities and mechanisms can be naturally described in the biological contexts [27]. Our approach could systematically identify BC-related circRNA modules relying on relatively complete disease information of mRNAs, miRNAs and pathways, which may provide more confident knowledge for the further identification of BC candidate circRNAs. Through integration analysis, thirteen circRNA modules, containing 4164 nodes (2703 genes, 80 circRNAs, 63 miRNAs and 1318 pathways) and 67,959 edges, were identified by our approach. Among them, we found 31 circRNAs in eight modules that closely related to known BC-related genes, miRNAs or pathways, which might be associated with the development and progression of BC. These identified circRNA modules could provide more insights into the investigation of their functional mechanisms, and will benefit the illumination of circRNA functions for clinical applications for BC patients in the future. To our knowledge, we first applied the NMF method to identify BC-related circRNA modules through the integration of multiple omics data from mRNAs, miRNAs and pathways, which largely facilitated the efficient prioritization and identification of BC candidate circRNAs and provided the potential circRNA biomarkers for the clinical diagnosis and treatment in BC patients.

## 2. Results

### 2.1. Identification of Differentially Expressed circRNAs and miRNAs in BC

**Figure 1:**
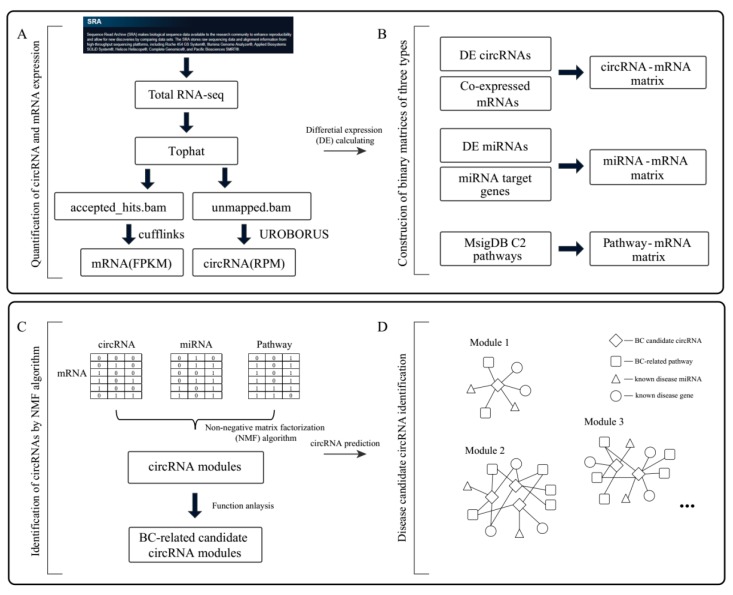
The flowchart of identification of breast cancer (BC)-related circRNA modules. The flowchart depicted a summary of the most important steps of the analysis workflow.

In total, there are 8953 human circRNAs recorded in the circBase database (http://www.circbase.org/) were identified from the RNA-seq data of BC patients by using the UROBORUS tool (Figure 1A). Then, 854 circRNAs expressed in at least 50% of patients were retained. Since several studies have shown that differentially expressed circRNAs or miRNAs would like to be associated with the disease with high probabilities [28,29], 80 differential expression circRNAs (four upregulated and 76 downregulated) with fold change (FC) values >2 or <0.5 were gathered (Supplementary Table S1). And 63 miRNAs that differently expressed (FC values >2 or <0.5 and Wilcoxon signed rank *p* < 0.005) in at least 50% of BC patient samples were retained for the following analysis.

Differentially expressed circRNAs were used to construct the circRNA-mRNA co-expression relations by calculating the Pearson correlation coefficient (PCC) values (Figure 1B). In total, 80 circRNAs and 17,519 mRNAs associated with 124,486 co-expressed pairs (PCC > 0.4 and *p* < 0.05) were obtained. Further, 80 circRNAs and 13,251 mRNAs with degrees more than three (119,528 co-expressed circRNA-mRNA pairs) were selected and were used to characterize the circRNA-mRNA binary matrix. MiRNA and mRNA relationships were integrated by the miRNA-target gene data, which were collected from starBase, miRTarBase, and PITA. 63 miRNAs and 8385 mRNAs with more than three partners were retained and were used to construct the miRNA-mRNA binary matrix. The pathway-mRNA relations were integrated from pathway data obtained from the Molecular Signatures Database (http://software.broadinstitute.org/gsea/msigdb), which contained a large number of functional annotation information that was curated from BioCart, Kyoto Encyclopedia of Genes and Genomes (KEGG), the NCI Pathway Interaction Database (PID), and Reactome. Finally, 1329 pathways and relevant 8904 mRNAs were used to characterize mRNA-pathway binary matrix. Three characterized binary matrices in total contained 2703 common mRNAs, 63 miRNAs, 80 circRNAs and 1318 pathways (Table 1).

### 2.2. Identification of circRNA Modules Based on a Non-Negative Matrix Factorization (NMF) Algorithm

The NMF algorithm was previously shown to be a useful decomposition method for multivariate data, in which the existing features can be transformed into a lower dimensional space. This algorithm can be applied to many practical problems in bioinformatics and computational biology such as integration analysis of different data. Therefore, based on three binary matrices of circRNA-mRNA, miRNA-mRNA and pathway-mRNA, we used the NMF algorithm to identify modules that were more representative and associated with BC-related functions. When *K* (the default parameter ranges from 5 to 20) equals to 13, the value of objective function *F* reached the minimum Euclidean error and the corresponding 13 circRNA modules were generated, including 4164 nodes (80 circRNAs, 2703 genes, 63 miRNAs and 1318 pathways) and 67,959 edges. Subsequently, 9 circRNA modules (Table 2) having more than 10 GO biological process (BP) functional categories were retained as BC-related circRNA modules (see details in Methods and Materials), including 1174 mRNAs, 44 circRNAs, 30 miRNAs and 325 pathways.

**Figure 2:**
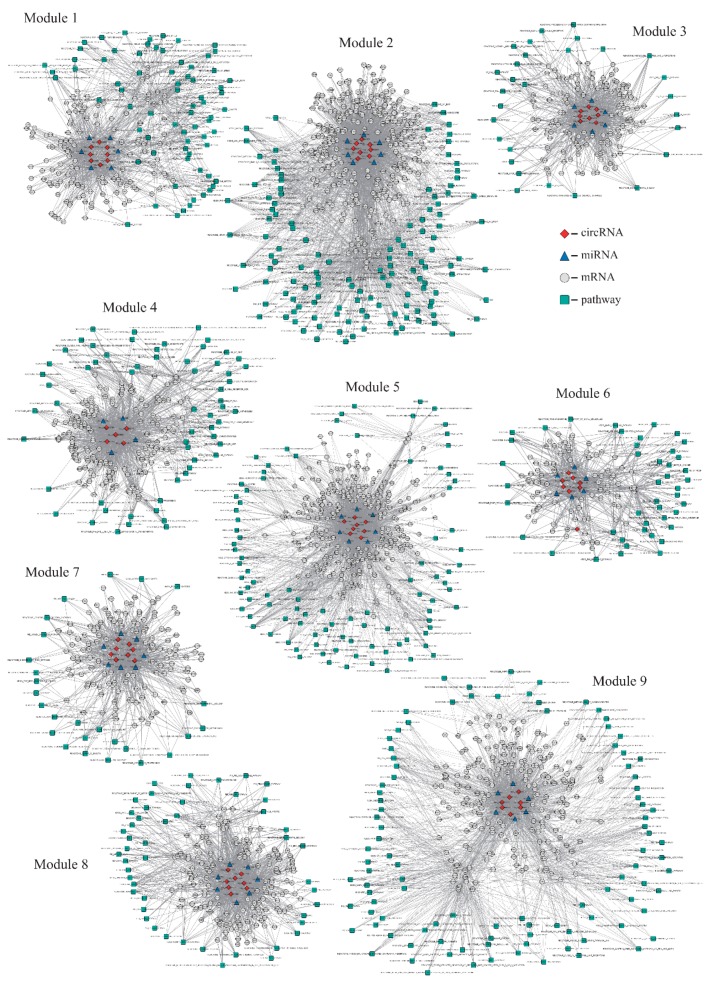
The overview of nine circRNA functional modules, including 1174 mRNAs, 44 circRNAs, 30 miRNAs and 325 pathways.

Modules 1 to 9 contained 222, 415, 172, 233, 382, 141, 171, 216 and 331 nodes (circRNAs, mRNAs, miRNAs or pathways), and 1069, 3299, 864, 1375, 2708, 665, 827, 1237 and 2054 edges, respectively (Figure 2 and Table 2). Within them, hsa_circ_0006528 in module 1 and module 3 has been validated to be related to BC [30]. There was a common gene named DDX3X in five modules including module 1, 3, 6, 7 and 8. DDX3X was abnormally expressed in breast epithelial cancer cells in which its expression was activated by HIF1A during hypoxia. Meanwhile, eight known BC-related genes—AKT1, CHEK2, ERBB2, PIK3CA, PPM1D, PTEN, SMAD4 and TSG101—were found in the nine modules (Supplementary Table S2). Twenty known BC-related miRNAs were also found: hsa-mir-7, hsa-let-7f, hsa-mir-103a, hsa-mir-130b, hsa-mir-135a, hsa-mir-144, hsa-mir-146a, hsa-mir-182, hsa-mir-185, hsa-mir-190a, hsa-mir-200a, hsa-mir-204, hsa-mir-216b, hsa-mir-224, hsa-mir-26a, hsa-mir-34b, hsa-mir-374b, hsa-mir-378a, hsa-mir-449a and hsa-mir-625 were also found in these modules (Supplementary Table S2). 

To better characterize the relationships between circRNAs and pathways or miRNAs in the nine modules, the normalized term overlap (NTO) scores [31] were calculated for each candidate circRNA-pathway pair and circRNA-miRNA pair (see details in Methods and Materials). Then, 44 circRNAs and 20 BC-related miRNA with high similar relations (NTO ≥ 0.5) were obtained (Supplementary Table S3). Also, 44 circRNAs and 14 KEGG pathways with high similar relations (NTO ≥ 0.5) were obtained (Supplementary Table S4), including ‘KEGG PATHWAYS IN CANCER’, ‘REACTOME IMMUNE SYSTEM’, ‘KEGG MAPK SIGNALING PATHWAY’, ‘KEGG CALCIUM SIGNALING PATHWAY’, ‘KEGG PROSTATE CANCER’, ‘PID ERBB1 DOWNSTREAM PATHWAY’, ‘PID P53 DOWNSTREAM PATHWAY’, ‘KEGG GNRH SIGNALING PATHWAY’, ‘KEGG P53 SIGNALING PATHWAY’, ‘KEGG SMALL CELL LUNG CANCER’, ‘KEGG WNT SIGNALING PATHWAY’, ‘PID CXCR4 PATHWAY’, ‘PID NOTCH PATHWAY’ and ‘REACTOME ACTIVATED TLR4 SIGNALLING’. Most of these pathways were cancer-related, showing that these circRNA-related modules in the nine modules may play important functional roles during the BC development and progression.

### 2.3. Prediction of Disease Candidate circRNAs in BC

To further identify candidate circRNAs that may be potentially associated with BC patients, circRNAs having more than four direct interaction partners (known BC genes, miRNAs or pathways, NTO score ≥ 0.5) were extracted (Supplementary Tables S2–S4, see details in Methods and Materials). Then, 31 unique candidate BC circRNAs were identified from module 1 to 9 (no circRNA in module 7), including 1, 8, 2, 5, 7, 4, 1 and 5 BC candidate circRNAs, respectively. Most of these circRNAs were associated with BC known disease genes (AKT1, PIK3CA, PPM1D, SMAD4 and TSG101) or miRNAs (hsa-let-7f, hsa-mir-7, hsa-mir-103a, hsa-mir-135a, hsa-mir-144, hsa-mir-146a, hsa-mir-182, hsa-mir-185, hsa-mir-190a, hsa-mir-204, hsa-mir-216b, hsa-mir-26a, hsa-mir-374b, hsa-mir-378a, hsa-mir-449a and hsa-mir-625). Especially, 30 out 31 BC candidate circRNAs: hsa_circ_0001222, hsa_circ_0002886, hsa_circ_0004458, hsa_circ_0004575, hsa_circ_0004910, hsa_circ_0007895, hsa_circ_0027842, hsa_circ_0079753 in module 2, hsa_circ_0002138, hsa_circ_0003614, hsa_circ_0003638, hsa_circ_0007766 in module 4, hsa_circ_0001725, hsa_circ_0007843, hsa_circ_0008362, hsa_circ_0017242, hsa_circ_0069244, hsa_circ_0073901, hsa_circ_0086375 in module 5, hsa_circ_0001558, hsa_circ_0017924, hsa_circ_0044177, hsa_circ_0069492 in module 6, hsa_circ_0001119, hsa_circ_0004513, hsa_circ_0007785, hsa_circ_0020399, hsa_circ_0037130 in module 9, hsa_circ_0003759 in module 3 and 4, hsa_circ_0001447 in module 3 and 8 were related to ‘KEGG PATHWAYS IN CANCER’, ‘REACTOME IMMUNE SYSTEM’ and ‘KEGG MAPK SIGNALING PATHWAY’, ‘KEGG P53 SIGNALING PATHWAY’ or ‘KEGG WNT SIGNALING PATHWAY’, which were closely involved with BC.

Interestingly, several circRNAs’ corresponding parental genes, such as has_circ_0007766, has_circ_0017242, has_circ_0037130, has_circ_0003759, has_circ_0007843, has_circ_0086375 and has_circ_0003614, were respectively recorded as ERBB2, AKT3, NPRL3, LPP, ARHGAP32, NFIB and ASPH in the circBase database. All these genes were remarked as ‘Cancer-related genes’ or ‘Disease related genes’ in the Human Protein Atlas (https://www.proteinatlas.org/), which also suggested that these circRNAs may be potentially served as disease biomarkers for disease diagnosis or therapy during disease development or progression.

### 2.4. Comparison with Other circRNA Prioritization Approaches

To further evaluate the performance of our approach in the identification of BC-related circRNA modules, we compared the circRNA modules generated by our approach with those yielded by the MCL algorithm. MCL is a traditional cluster method for networks, which has been widely used for clustering of genes, proteins or other biomarkers according to their expression profile or other experimentally detected data [32,33]. According to the same comprehensive network, the MCL algorithm was performed by the Cytoscape plugin clusterMaker (the minimum number of nodes in each module was set to 20). Then, 8 modules (Supplementary Table S5 and Supplementary Figure S1) were generated, of which module 1 was the biggest one with 1678 nodes, and module 2 to 7 contained 60, 40, 36, 25, 22 and 20 nodes, respectively. Due to the different sizes of circRNA modules and the different nodes in each modules generated by MCL and our approach, it was impossible to directly compare the results of these two approaches in the identification of BC-related circRNAs. Thus, we indirectly compared circRNA modules obtained from these two approaches by statistic proportion of nodes with known BC information. We found BC known disease circRNAs in our module 1 and 3 account for 0.45% and 0.58%, and in MCL module 1 account for 0.12%. Other nodes (include genes, miRNAs and pathways) with known BC information in circRNA modules of our approach account for 5.405%, 2.651%, 4.651%, 3.433%, 3.141%, 8.511%, 3.509%, 4.167% and 2.720% while 1.728%, 0, 0, 0, 0, 0, and 5% in MCL circRNA modules, respectively (Supplementary Table S5). The comparison results suggested that our approach identified circRNA modules with more BC-related information, which could be able to capture more characterization about BC. Furthermore, GO enrichment analysis suggested that more functional GO terms were enriched in modules generated by our approach (R-package ‘clusterProfiler’, Benjamini–Hochberg correction, FDR < 0.05), which demonstrated that our circRNA modules were more closely related to the development and progression of BC (Supplementary Table S5).

## 3. Discussion

Increasing numbers of disease-related molecular biomarkers, including gene, protein, miRNA, lncRNA, and circRNA could provide more promises to improve the diagnosis and treatment for BC patients [34,35,36]. However, relatively incomplete disease information about circRNA brings a challenge to biological researchers to uncover their functional mechanisms and roles. Towards this end, we developed a computational pipeline with the goal of identifying BC-related circRNA modules by integration of circRNA, mRNA, miRNA, and pathway data based on an NMF algorithm in this work. Our approach integrated known disease information and omics data, whereby we could identify BC candidate circRNAs and infer their functional roles.

Employing the systemic pipeline in 33 BC RNA-seq data with tumor and normal samples, we identified 13 circRNA modules in BC, containing 80 circRNAs, 2703 genes, 63 miRNAs and 1318 pathways with 6,795,944 interactions. After screening by functional enrichment analysis, nine circRNA modules potentially associated with BC were obtained. Within them, one circRNA hsa_circ_0006528 had been recognized as known disease circRNA. Simultaneously, eight genes and twenty miRNAs in circRNA modules have been validated as known BC biomarkers. Functional enrichment results showed that other 31 circRNAs were closely related with known disease miRNA or BC associated pathways. The circRNA prioritization result of our approach suggested that known disease information curated by circRNA direct partners, including genes, miRNAs and pathways, could give more chances to recognize disease related circRNAs. Comparison with other module identification methods like MCL, NMF algorithm identified more BC informative circRNA modules and could comprehensively characterize BC from circRNA perspective.

There are some limitations to our approach. Relative small numbers of BC candidate circRNAs were included and analyzed in our approach, which limited the ability of our approach in the prediction of circRNA’s functions in human BC context. In addition, relatively strict screening criteria were adopted in functional modules identification, which tended to remove some meaningful circRNA modules. Thus, there are also some other proposed methods for the identification of disease-related factors and modules which could be used for reference [37,38,39]. For example, Chen X et al. developed decision tree learning-based model (EGBMMDA) for predicting miRNA-disease associations, by integrating the miRNA functional similarity, the disease semantic similarity, and known miRNA–disease associations [40]. BC-related circRNAs would be efficiently identified by this method if more functional categories are explicit for circRNAs. 

In summary, we proposed a systematic approach to identify BC-related circRNA modules through the NMF algorithm. These identified circRNA modules provide novel insights into the potentially BC-associated circRNAs, which will benefit the clinical applications of circRNA biomarkers for BC diagnosis, treatment and prognosis in the future.

## 4. Materials and Methods

### 4.1. Data Acquisitions

Paired-end RNA-seq data of SRP062132, which detected by “Illumina Genome Analyzer II” were downloaded from the NCBI SRA database (https://www.ncbi.nlm.nih.gov/Traces/study/?acc=SRP062132&go=go). This dataset included 15 disease samples and 18 normal samples (Figure 1A). The miRNA expression profile data, GSE83270, which detected by ‘Exiqon miRCURY LNA microRNA array, 7th generation’ (GPL22003) for 12 BC patients, including 12 BC patients, was downloaded from the GEO database. The corresponding miRNA target genes were obtained from starBase [41] (http://starbase.sysu.edu.cn/), miRTarBase [42] (http://mirtarbase.mbc.nctu.edu.tw/php/index.php) and PITA (https://genie.weizmann.ac.il/pubs/mir07/mir07_data.html). Pathway data were downloaded from the Molecular Signatures Database [43] (http://software.broadinstitute.org/gsea/msigdb) database, which contained a large number of annotated functional genes collected from existing public databases, such as BioCart, KEGG, PID, and Reactome. We selected pathway data from the curated gene sets (c2) in MsigDB V6.1, which contained a total of 1329 metabolic and signaling pathways.

### 4.2. Quantification of circRNA-mRNA, miRNA-mRNA, and Pathway-mRNA Binary Matrices

For each sample of BC patients in SRP062132, the RNA-seq reads were first mapped to the human reference genome (GRCh37/hg19, UCSC Genome Browser [44]) by the TopHat2 [45] tool, which was capable of detecting the canonical splicing event (Figure 1A). In addition, the unmapped reads were then used to identify the circRNAs by the pipeline proposed by UROBORUS [46]. During the process of quantification of human circRNAs, the unmapped reads were extracted to 20-bp anchors from head ends and tail ends. The short 20-bp paired-end seed sequence reads were aligned to the human reference genome (hg19) with a maximum of 2 bp mismatches using TopHat2 with a default parameter value. Balanced mapped junction (BMJ) reads and unbalanced mapped junction (UMJ) reads were generated as two sets spanning the spliced site. BMJ or UMJ reads were represented as reads aligned to the joining region of two back spliced exons with minimum 20 bp of overhang at any an end or with less than 20 bp of overhang at one end, respectively. To evaluate the relative expression of circRNAs in different disease and normal tissues, we normalized the number of circular reads to per kilobase per million reads sequenced (RPKM) values. To quantify the expression levels of mRNAs, we used Cufflinks [47] software to process the accepted hits.bam file in the TopHat2 results, which contained all reads mapped to the human reference genome. We also used RPKM value to identify the relative expression of each mRNA.

After recognized by the UROBORUS, circRNAs recorded in the circBase database and expressed in more than 50% patient samples were retained (Figure 1A). The differentially expressed circRNAs with FC values >2 or <0.5 were identified. In GSE83270, miRNAs with FC >2 or <0.5 and Wilcoxon signed rank test *p* < 0.005 were considered as differentially expressed miRNAs. The PCC was then used to measure the co-expression relationships between differentially expressed circRNAs and mRNAs. CircRNA-mRNA pairs with PCC > 0.4 and *p*-value < 0.05 were used to construct the binary matrix. As for the miRNAs and mRNAs, the corresponding miRNA target mRNAs relations were used to build the miRNA-mRNA binary matrix. If a pair of miRNA and mRNA was recorded in any one of the three database starBase, miRTarBase and PITA, the miRNA and mRNA was denoted as “1” in the miRNA-mRNA binary matrix. The pathway-mRNA binary matrix was similarly constructed based on pathway information from the Molecular Signatures Database.

### 4.3. Construction of circRNA Modules Basing on Non-Negative Matrix Factorization (NMF) Algorithm

We identified different numbers *K* of BC-related functional modules from these three matrices by using non-negative matrix factorization (NMF). The objective function *F* for NMF was defined as:

(1) $$F(W,H)=\sum_{I=1}^{3}{\left\|X_{I}-WH_{I}\right\|}^{2}$$

where $X_{I\;}\left(I\in \left(1,2,3\right)\right)$ represented the characterized binary circRNA-mRNA, miRNA-mRNA, and pathway-mRNA matrix, respectively. The same penalization parameters for characterization of binary circRNA-mRNA, miRNA-mRNA, and pathway-mRNA matrices were assigned as described in Liu’s NMF approaches [26], and the penalization parameters were set as default zero. *W* and *H* were both non-negative matrices. *W* was an *M* × *K* (*M* was the number of common mRNAs in three matrices) matrix representing the basis vector. $H_{I}\;\left(I\in \left(1,2,3\right)\right)$ was a *K* × *N* (*N* is the numbers of circRNAs, miRNAs, and pathways) matrix, representing the coefficient vector in the dimension reduction process (Figure 1C). We selected different *K* (from 5 to 20) numbers and calculated the Euclidean errors between the input matrices, and the model reconstructed data. The Euclidean error measured the distance between the input matrices and the model reconstructed data. By comparing the Euclidean errors, we selected the smallest one to build the functional modules. *W* and *H* were updated at each iteration step by using the generalized multiplicative update rules as follows:

(2) $$W_{ij}=W_{ij}\frac{{\left(X_{1}H_{1}{}^{T}+X_{2}H_{2}{}^{T}+X_{3}H_{3}{}^{T}\right)}_{ij}}{{\left(W(H_{1}H_{1}{}^{T}+H_{2}H_{2}{}^{T}+H_{3}H_{3}{}^{T})\right)}_{ij}}$$

(3) $${\left(H_{I}\right)}_{ij}={\left(H_{I}\right)}_{ij}\frac{{\left(W^{T}X_{I}\right)}_{ij}}{{\left(W^{T}WH_{I}\right)}_{ij}},I=1,2,3.$$

It was worth noting that when we used the randomly generated initial matrices *W* and *H_I_* (*I* ∈ (1,2,3)) to minimize the Euclidean distance function, a local minimum solution occasionally appeared. We thus repeated the optimization procedure 100 times with random initial solution matrices to address this limitation. The lowest object function value was selected as the final factorization solution, and the selected value *K* meant that we finally got *K* modules. Then the obtained decomposing matrices *W* and *H_I_* (*I* ∈ (1,2,3)) were normalized through Z-score normalization by the following formula:

(4) $$z_{ij}=\frac{x_{ij}-\mu_{i}}{\sigma_{i}}$$

where $\mu_{i}$ represented the mean value of elements in the *i-th* column of *W* matrix or in the *i-th* row of *H_I_* (*I* ∈ (1,2,3)) matrix, and $\sigma_{i}$ was the corresponding standard deviation. The obtained Z-score values were used to determine each module members (including mRNAs, miRNAs, circRNAs and pathways) according to a published method [48]. For each column of matrix *W* (corresponding to an identified module), we separately retrieved the top 1% to top 10% ranked mRNAs according to the Z-score values to perform the GO BP enrichment analysis, by using R-package ‘clusterProfiler’ (Benjamini–Hochberg correction, FDR < 0.05). Then, when the top k% mRNAs enriched the most BP GO terms, we assigned the top k% genes to this module. Similarly, we assigned the top k% miRNAs, circRNAs or pathways in the corresponding row of matrix *H_I_* (*I* ∈ (1,2,3)) to the same module (Figure 1C). Subsequently, disease candidate circRNAs were identified according to the relationships between circRNAs and known BC genes, miRNAs or BC-related pathways in these functional modules (Figure 1D). Those circRNAs having more than four direct interaction partners (known BC genes, miRNAs or pathways) were identified as BC relative circRNAs. The known BC genes and miRNAs were obtained from GeneCards (https://www.genecards.org/) and HMDD (http://www.cuilab.cn/hmdd) respectively.

In addition, we calculated the normalized term overlap (NTO) score to further determine the relationships between circRNAs and pathways or miRNAs. The NTO score was calculated by the formula as follows:

(5) $$NTO=\frac{\left|E_{G}\cap E_{T}\right|}{\min(\left|E_{G}\right|,\left|E_{T}\right|)}$$

where $E_{G}$ represented the number of associated mRNAs for a specific circRNA, $E_{T}$ represented the number of mRNAs associated with a pathway or miRNA, $\left|E_{G}\cap E_{T}\right|$ represents the number of common mRNAs for circRNAs and pathways or miRNAs, and $\min(\left|E_{G}\right|,\left|E_{T}\right|)$ represented the minimum numbers of mRNAs of circRNAs and pathways or miRNAs. The above processing was implemented using the R software environment.

## Figures and Tables

**Table 1:** Summary information of three characterized binary matrixes.

Association Matrix	#(circRNA/miRNA/patnway)	#(mRNA)	Dimensions
circRNA-mRNA matrix	80	2703	80 × 2703
miRNA-mRNA matrix	63	2703	63 × 2703
pathway-mRNA matrix	1318	2703	1318 × 2703

**Table 2:** Summary of 9 circRNA modules, including 2703 genes, 80 circRNAs, 63 miRNAs and 1318 pathways.

Modules	Nodes	CircRNAs	mRNAs	miRNAs	Pathways	Edges
1	222	8	136	6	72	1069
2	415	8	271	7	129	3299
3	172	8	129	6	29	864
4	233	5	163	4	61	1375
5	382	8	271	7	96	2708
6	141	8	83	6	44	665
7	171	8	130	7	26	827
8	216	8	152	7	49	1237
9	331	7	217	6	101	2054

## Supplementary Materials

The following are available online at http://www.mdpi.com/1422-0067/20/4/919/s1.
