# Supplementary material for: Systematical Identification of Breast Cancer-Related Circular RNA Modules for Deciphering circRNA Functions Based on the Non-Negative Matrix Factorization Algorithm
Source: Int J Mol Sci. 2019 Feb 20;20(4):919. doi: 10.3390/ijms20040919 (PMC6412941; doi:10.3390/ijms20040919)
Supplement: Supplementary file 1 [file ijms-20-00919-s001.zip › Supplementary Files/Supplementary Table s1.docx]

**Supplementary Table 1. 80 differentially expressed circRNAs and their FC values**

| circRNA id | chromosome | Fold Change |
| --- | --- | --- |
| hsa_circ_0007766 | chr17 | 2.11 |
| hsa_circ_0020399 | chr10 | 2.27 |
| hsa_circ_0000034 | chr1 | 2.17 |
| hsa_circ_0007308 | chr4 | 3.04 |
| hsa_circ_0037130 | chr16 | 0.42 |
| hsa_circ_0003614 | Chr8 | 0.46 |
| hsa_circ_0004513 | Chr12 | 0.49 |
| hsa_circ_0009964 | Chr1 | 0.48 |
| hsa_circ_0004910 | Chr16 | 0.38 |
| hsa_circ_0003273 | Chr2 | 0.49 |
| hsa_circ_0002886 | Chr12 | 0.41 |
| hsa_circ_0028094 | Chr12 | 0.42 |
| hsa_circ_0001119 | Chr2 | 0.41 |
| hsa_circ_0001558 | Chr5 | 0.49 |
| hsa_circ_0006608 | Chr10 | 0.41 |
| hsa_circ_0004539 | Chr5 | 0.48 |
| hsa_circ_0004390 | Chr1 | 0.33 |
| hsa_circ_0004412 | Chr1 | 0.49 |
| hsa_circ_0008362 | Chr10 | 0.47 |
| hsa_circ_0007785 | Chr13 | 0.37 |
| hsa_circ_0008550 | Chr3 | 0.48 |
| hsa_circ_0084143 | Chr8 | 0.48 |
| hsa_circ_0006893 | Chr3 | 0.43 |
| hsa_circ_0001725 | Chr7 | 0.47 |
| hsa_circ_0000198 | Chr1 | 0.42 |
| hsa_circ_0034293 | Chr15 | 0.46 |
| hsa_circ_0075796 | Chr6 | 0.36 |
| hsa_circ_0001696 | Chr7 | 0.44 |
| hsa_circ_0003638 | Chr17 | 0.48 |
| hsa_circ_0018168 | Chr10 | 0.49 |
| hsa_circ_0002501 | Chr18 | 0.43 |
| hsa_circ_0089973 | chrX | 0.47 |
| hsa_circ_0001369 | Chr3 | 0.47 |
| hsa_circ_0001447 | Chr4 | 0.36 |
| hsa_circ_0001350 | Chr3 | 0.36 |
| hsa_circ_0036627 | Chr15 | 0.48 |
| hsa_circ_0001681 | Chr7 | 0.36 |
| hsa_circ_0017242 | Chr1 | 0.45 |
| hsa_circ_0079753 | Chr7 | 0.33 |
| hsa_circ_0086375 | Chr9 | 0.39 |
| hsa_circ_0008836 | Chr5 | 0.49 |
| hsa_circ_0005199 | Chr1 | 0.29 |
| hsa_circ_0002138 | Chr15 | 0.43 |
| hsa_circ_0006528 | Chr5 | 0.29 |
| hsa_circ_0006215 | Chr3 | 0.35 |
| hsa_circ_0002473 | Chr13 | 0.44 |
| hsa_circ_0069244 | Chr4 | 0.31 |
| hsa_circ_0005567 | Chr1 | 0.48 |
| hsa_circ_0007324 | Chr4 | 0.41 |
| hsa_circ_0002918 | Chr2 | 0.44 |
| hsa_circ_0004719 | Chr17 | 0.46 |
| hsa_circ_0001358 | Chr3 | 0.38 |
| hsa_circ_0008759 | Chr3 | 0.42 |
| hsa_circ_0004956 | Chr19 | 0.48 |
| hsa_circ_0000824 | Chr18 | 0.49 |
| hsa_circ_0073901 | Chr5 | 0.19 |
| hsa_circ_0069492 | Chr4 | 0.48 |
| hsa_circ_0086376 | Chr9 | 0.49 |
| hsa_circ_0004458 | Chr8 | 0.42 |
| hsa_circ_0044177 | Chr17 | 0.32 |
| hsa_circ_0007843 | Chr11 | 0.49 |
| hsa_circ_0004575 | Chr2 | 0.40 |
| hsa_circ_0004327 | Chr6 | 0.34 |
| hsa_circ_0007822 | Chr1 | 0.47 |
| hsa_circ_0001993 | Chr18 | 0.32 |
| hsa_circ_0007895 | Chr1 | 0.47 |
| hsa_circ_0017924 | Chr10 | 0.47 |
| hsa_circ_0020238 | Chr10 | 0.46 |
| hsa_circ_0003759 | Chr3 | 0.42 |
| hsa_circ_0003571 | Chr5 | 0.39 |
| hsa_circ_0008027 | Chr3 | 0.42 |
| hsa_circ_0045537 | Chr17 | 0.43 |
| hsa_circ_0000982 | Chr2 | 0.45 |
| hsa_circ_0027842 | Chr12 | 0.36 |
| hsa_circ_0045308 | Chr17 | 0.46 |
| hsa_circ_0081207 | Chr7 | 0.43 |
| hsa_circ_0004313 | Chr12 | 0.23 |
| hsa_circ_0001222 | Chr22 | 0.48 |
| hsa_circ_0067717 | Chr3 | 0.48 |
| hsa_circ_0007167 | Chr1 | 0.46 |
