# Supplementary material for: Systematical Identification of Breast Cancer-Related Circular RNA Modules for Deciphering circRNA Functions Based on the Non-Negative Matrix Factorization Algorithm
Source: Int J Mol Sci. 2019 Feb 20;20(4):919. doi: 10.3390/ijms20040919 (PMC6412941; doi:10.3390/ijms20040919)
Supplement: Supplementary file 1 [file ijms-20-00919-s001.zip › Supplementary Files/Supplementary Table s3.docx]

**Supplementary Table 3. The NTO score of circRNA and corresponding disease miRNA.**

| Modules | circRNAs | miRNA | NTO score |
| --- | --- | --- | --- |
| Module 1 | hsa_circ_0006893 | hsa-mir-103a  hsa-mir-200a | 1  0.9 |
|  | hsa_circ_0006528 | hsa-mir-103a  hsa-mir-200a  hsa-mir-204  hsa-mir-135a | 1  0.75  0.5625  0.5625 |
|  | hsa_circ_0008836 | hsa-mir-103a  hsa-mir-200a  hsa-mir-204  hsa-mir-135a | 1  0.75  0.5625  0.5625 |
|  | hsa_circ_0005567 | hsa-mir-103a  hsa-mir-200a  hsa-mir-204 | 0.9722  0.7222  0.5278 |
|  | hsa_circ_0001558 | hsa-mir-103a  hsa-mir-200a | 0.9714  0.9143 |
|  | hsa_circ_0001696 | hsa-mir-103a  hsa-mir-200a | 0.9476  0.9143 |
|  | hsa_circ_0002473 | hsa-mir-103a  hsa-mir-200a  hsa-mir-204 | 0.9412  0.7647  0.5882 |
|  | hsa_circ_0075796 | hsa-mir-103a  hsa-mir-200a  hsa-mir-204 | 0.9375  0.9375  0.5625 |
| Module 2 | hsa_circ_0004575 | hsa-mir-144  hsa-mir-190a  hsa-mir-185  hsa-mir-135a | 0.9912  0.9872  0.9808  0.9722 |
|  | hsa_circ_0004458 | hsa-mir-135a  hsa-mir-185  hsa-mir-190a  hsa-mir-144 | 0.875  0.9038  0.8462  0.823 |
|  | hsa_circ_0007895 | hsa-mir-190a  hsa-mir-185  hsa-mir-135a  hsa-mir-144 | 0.8718  0.8654  0.8611  0.8496 |
|  | hsa_circ_0002886 | hsa-mir-190a  hsa-mir-185  hsa-mir-135a  hsa-mir-144 | 0.859  0.8462  0.8333  0.823 |
|  | hsa_circ_0027842 | hsa-mir-190a  hsa-mir-144  hsa-mir-135a  hsa-mir-185 | 0.8205  0.7965  0.7778  0.7380 |
|  | hsa_circ_0001222 | hsa-mir-135a  hsa-mir-185  hsa-mir-144  hsa-mir-190a | 0.7639  0.75  0.6991  0.6923 |
|  | hsa_circ_0079753 | hsa-mir-185  hsa-mir-144  hsa-mir-190a  hsa-mir-135a | 0.7885  0.7522  0.7308  0.7361 |
|  | hsa_circ_0004910 | hsa-mir-185  hsa-mir-135a  hsa-mir-144  hsa-mir-190a | 0.6731  0.6667  0.6195  0.6026 |
| Module 3 | hsa_circ_0001447 | hsa-mir-144  hsa-mir-374b  hsa-mir-26a | 1  0.9318  0.6364 |
|  | hsa_circ_0001696 | hsa-mir-144  hsa-mir-26a  hsa-mir-374b | 1  0.7857  0.7143 |
|  | hsa_circ_0008759 | hsa-mir-144  hsa-mir-374b  hsa-mir-26a  hsa-mir-224 | 1  0.9231  0.5385  0.5385 |
|  | hsa_circ_0002473 | hsa-mir-144  hsa-mir-374b  hsa-mir-26a  hsa-mir-224 | 1  0.9286  0.5714  0.5 |
|  | hsa_circ_0006528 | hsa-mir-144  hsa-mir-374b  hsa-mir-26a | 1  0.913  0.6087 |
|  | hsa_circ_0003759 | hsa-mir-144  hsa-mir-374b  hsa-mir-26a  hsa-mir-135a  hsa-mir-224  hsa-mir-7 | 0.9848  0.9394  0.6364  0.5435  0.5102  0.5 |
|  | hsa_circ_0069492 | hsa-mir-144  hsa-mir-374b  hsa-mir-26a  hsa-mir-224 | 0.96  0.96  0.64  0.52 |
|  | hsa_circ_0075796 | hsa-mir-144  hsa-mir-374b  hsa-mir-26a  hsa-mir-7  hsa-mir-135a | 0.8571  0.8571  0.8571  0.5  0.5 |
| Module 4 | hsa_circ_0007766 | hsa-mir-378a  hsa-mir-7  hsa-mir-146a | 1  1  1 |
|  | hsa_circ_0003614 | hsa-mir-378a  hsa-mir-7  hsa-mir-146a | 1  1  1 |
|  | hsa_circ_0002138 | hsa-mir-146a  hsa-mir-378a  hsa-mir-7 | 0.9231  0.9216  0.9 |
|  | hsa_circ_0003638 | hsa-mir-378a  hsa-mir-7  hsa-mir-146a | 0.925  0.8627  0.8462 |
|  | hsa_circ_0003759 | hsa-mir-146a  hsa-mir-378a  hsa-mir-7 | 0.8846  0.8039  0.75 |
| Module 5 | hsa_circ_0069244 | hsa-mir-625  hsa-mir-204  hsa-mir-146a  hsa-let-7f | 0.9286  0.8491  0.8261  0.7419 |
|  | hsa_circ_0017242 | hsa-mir-146a  hsa-mir-625  hsa-let-7f  hsa-mir-204 | 0.9348  0.9286  0.871  0.8491 |
|  | hsa_circ_0007843 | hsa-mir-204  hsa-mir-625  hsa-let-7f  hsa-mir-146a | 0.8113  0.7857  0.7581  0.7174 |
|  | hsa_circ_0001725 | hsa-mir-204  hsa-mir-146a  hsa-let-7f  hsa-mir-625 | 0.7925  0.7609  0.7259  0.5714 |
|  | hsa_circ_0084143 | hsa-mir-204  hsa-let-7f  hsa-mir-146a  hsa-mir-625 | 0.7358  0.6774  0.6740  0.5714 |
|  | hsa_circ_0086375 | hsa-mir-146a  hsa-mir-625  hsa-let-7f  hsa-mir-204 | 0.8913  0.7857  0.7742  0.7358 |
|  | hsa_circ_0073901 | hsa-mir-146a  hsa-mir-625  hsa-mir-204  hsa-let-7f | 0.6522  0.6429  0.6415  0.5323 |
|  | hsa_circ_0008362 | hsa-mir-625  hsa-mir-146a  hsa-mir-204  hsa-let-7f | 0.6429  0.6304  0.566  0.5 |
| Module 6 | hsa_circ_0004412 | hsa-mir-449a  hsa-mir-182 | 1  1 |
|  | hsa_circ_0000034 | hsa-mir-449a  hsa-mir-182 | 1  0.9167 |
|  | hsa_circ_0044177 | hsa-mir-449a  hsa-mir-182 | 0.9167  0.8333 |
|  | hsa_circ_0069492 | hsa-mir-449a  hsa-mir-182  hsa-mir-34b | 0.9167  0.75  0.5417 |
|  | hsa_circ_0001558 | hsa-mir-449a  hsa-mir-182 | 0.9032  0.7419 |
|  | hsa_circ_0017924 | hsa-mir-449a  hsa-mir-182 | 0.88  0.76 |
|  | hsa_circ_0001696 | hsa-mir-449a  hsa-mir-182 | 0.8462  0.6923 |
|  | hsa_circ_0006893 | hsa-mir-182  hsa-mir-449a | 0.9  0.8 |
| Module 7 | hsa_circ_0044177 | hsa-mir-130b | 1 |
|  | hsa_circ_0017924 | hsa-mir-130b | 1 |
|  | hsa_circ_0069492 | hsa-mir-130b | 1 |
|  | hsa_circ_0001350 | hsa-mir-130b | 1 |
|  | hsa_circ_0000034 | hsa-mir-130b | 1 |
|  | hsa_circ_0001558 | hsa-mir-130b | 1 |
|  | hsa_circ_0006893 | hsa-mir-130b | 1 |
|  | hsa_circ_0008759 | hsa-mir-130b | 1 |
| Module 9 | hsa_circ_0037130 | hsa-mir-216b  hsa-mir-374b  hsa-mir-374b  hsa-mir-7  hsa-mir-185 | 0.8889  0.8889  0.8667  0.8409  0.8302 |
|  | hsa_circ_0007785 | hsa-mir-216b  hsa-mir-374b  hsa-mir-7  hsa-mir-374b  hsa-mir-185 | 0.6889  0.6889  0.6591  0.6  0.5472 |
|  | hsa_circ_0020399 | hsa-mir-374b  hsa-mir-185  hsa-mir-216b  hsa-mir-374b  hsa-mir-7 | 0.7333  0.6981  0.6889  0.6889  0.5909 |
|  | hsa_circ_0004513 | hsa-mir-374b  hsa-mir-216b  hsa-mir-374b  hsa-mir-7  hsa-mir-185 | 0.6267  0.6  0.6  0.5682  0.5604 |
|  | hsa_circ_0001119 | hsa-mir-216b  hsa-mir-374b  hsa-mir-185  hsa-mir-374b  hsa-mir-7 | 0.5778  0.5778  0.585  0.5467  0.5227 |
|  | hsa_circ_0004539 | hsa-mir-7 | 0.5227 |
|  | hsa_circ_0006608 | hsa-mir-185  hsa-mir-7 | 0.5283  0.5 |
