# Supplementary material for: Systematical Identification of Breast Cancer-Related Circular RNA Modules for Deciphering circRNA Functions Based on the Non-Negative Matrix Factorization Algorithm
Source: Int J Mol Sci. 2019 Feb 20;20(4):919. doi: 10.3390/ijms20040919 (PMC6412941; doi:10.3390/ijms20040919)
Supplement: Supplementary file 1 [file ijms-20-00919-s001.zip › Supplementary Files/Supplementary Table s4.docx]

**Supplementary Table 4. The NTO score of circRNA and corresponding pathway.**

| Modules | CircRNAs | Pathways | NTO scores |
| --- | --- | --- | --- |
| Module 1 | hsa_circ_0008836 | KEGG_PROSTATE_CANCER  KEGG_PATHWAYS_IN_CANCER | 0.75  0.5 |
|  | hsa_circ_0005567 | KEGG_PROSTATE_CANCER | 0.5 |
| Module 2 | hsa_circ_0004575 | KEGG_CALCIUM_SIGNALING_PATHWAY  KEGG_PROSTATE_CANCER  PID_ERBB1_DOWNSTREAM_PATHWAY  KEGG_GNRH_SIGNALING_PATHWAY  PID_CXCR4_PATHWAY  REACTOME_IMMUNE_SYSTEM  KEGG_PATHWAYS_IN_CANCER  KEGG_MAPK_SIGNALING_PATHWAY | 1  1  1  1  1  0.9565  0.9565  0.9444 |
|  | hsa_circ_0002886 | KEGG_CALCIUM_SIGNALING_PATHWAY  KEGG_MAPK_SIGNALING_PATHWAY  KEGG_PROSTATE_CANCER  KEGG_PATHWAYS_IN_CANCER  KEGG_GNRH_SIGNALING_PATHWAY  REACTOME_IMMUNE_SYSTEM  PID_CXCR4_PATHWAY  PID_ERBB1_DOWNSTREAM_PATHWAY | 0.9231  0.8889  0.875  0.8696  0.8333  0.8276  0.7143  0.5833 |
|  | hsa_circ_0007895 | KEGG_GNRH_SIGNALING_PATHWAY  PID_CXCR4_PATHWAY  KEGG_CALCIUM_SIGNALING_PATHWAY  REACTOME_IMMUNE_SYSTEM  KEGG_PROSTATE_CANCER  KEGG_PATHWAYS_IN_CANCER  KEGG_MAPK_SIGNALING_PATHWAY  PID_ERBB1_DOWNSTREAM_PATHWAY | 1  1  0.9231  0.8966  0.875  0.8696  0.7778  0.6667 |
|  | hsa_circ_0027842 | KEGG_PROSTATE_CANCER  KEGG_GNRH_SIGNALING_PATHWAY  KEGG_PATHWAYS_IN_CANCER  KEGG_MAPK_SIGNALING_PATHWAY  KEGG_CALCIUM_SIGNALING_PATHWAY  PID_ERBB1_DOWNSTREAM_PATHWAY  REACTOME_IMMUNE_SYSTEM  PID_CXCR4_PATHWAY | 0.875  0.8333  0.8261  0.7778  0.7692  0.6667  0.5897  0.5714 |
|  | hsa_circ_0004458 | KEGG_PROSTATE_CANCER  REACTOME_IMMUNE_SYSTEM  KEGG_CALCIUM_SIGNALING_PATHWAY  KEGG_GNRH_SIGNALING_PATHWAY  KEGG_PATHWAYS_IN_CANCER  KEGG_MAPK_SIGNALING_PATHWAY  PID_ERBB1_DOWNSTREAM_PATHWAY  PID_CXCR4_PATHWAY | 1  0.8966  0.8462  0.8333  0.7826  0.7778  0.75  0.7143 |
|  | hsa_circ_0079753 | KEGG_PROSTATE_CANCER  PID_CXCR4_PATHWAY  KEGG_CALCIUM_SIGNALING_PATHWAY  PID_ERBB1_DOWNSTREAM_PATHWAY  KEGG_GNRH_SIGNALING_PATHWAY  KEGG_PATHWAYS_IN_CANCER  REACTOME_IMMUNE_SYSTEM  KEGG_MAPK_SIGNALING_PATHWAY | 0.875  0.8571  0.8462  0.8333  0.8333  0.7826  0.6897  0.6667 |
|  | hsa_circ_0001222 | KEGG_CALCIUM_SIGNALING_PATHWAY  PID_CXCR4_PATHWAY  KEGG_MAPK_SIGNALING_PATHWAY  PID_ERBB1_DOWNSTREAM_PATHWAY  KEGG_GNRH_SIGNALING_PATHWAY  REACTOME_IMMUNE_SYSTEM  KEGG_PATHWAYS_IN_CANCER  KEGG_PROSTATE_CANCER | 0.9231  0.8571  0.8333  0.8333  0.8333  0.7931  0.7826  0.75 |
|  | hsa_circ_0004910 | KEGG_GNRH_SIGNALING_PATHWAY  PID_CXCR4_PATHWAY  KEGG_PROSTATE_CANCER  KEGG_CALCIUM_SIGNALING_PATHWAY  PID_ERBB1_DOWNSTREAM_PATHWAY  KEGG_PATHWAYS_IN_CANCER  REACTOME_IMMUNE_SYSTEM  KEGG_MAPK_SIGNALING_PATHWAY | 1  1  1  0.8462  0.8333  0.7826  0.7586  0.6667 |
| Module 3 | hsa_circ_0003759 | KEGG_P53_SIGNALING_PATHWAY  PID_P53_DOWNSTREAM_PATHWAY  KEGG_PATHWAYS_IN_CANCER | 0.8333  0.6  0.6 |
|  | hsa_circ_0001447 | KEGG_P53_SIGNALING_PATHWAY | 0.5 |
| Module 4 | hsa_circ_0007766 | KEGG_PATHWAYS_IN_CANCER  REACTOME_IMMUNE_SYSTEM  KEGG_CALCIUM_SIGNALING_PATHWAY  PID_ERBB1_DOWNSTREAM_PATHWAY  PID_CXCR4_PATHWAY | 1  1  1  1  1 |
|  | hsa_circ_0003614 | KEGG_PATHWAYS_IN_CANCER  REACTOME_IMMUNE_SYSTEM  KEGG_CALCIUM_SIGNALING_PATHWAY  PID_ERBB1_DOWNSTREAM_PATHWAY  PID_CXCR4_PATHWAY | 1  1  1  1  1 |
|  | hsa_circ_0003638 | PID_CXCR4_PATHWAY  KEGG_CALCIUM_SIGNALING_PATHWAY  PID_ERBB1_DOWNSTREAM_PATHWAY  REACTOME_IMMUNE_SYSTEM  KEGG_PATHWAYS_IN_CANCER | 1  1  1  0.9231  0.9167 |
|  | hsa_circ_0003759 | PID_ERBB1_DOWNSTREAM_PATHWAY  KEGG_CALCIUM_SIGNALING_PATHWAY  REACTOME_IMMUNE_SYSTEM  PID_CXCR4_PATHWAY  KEGG_PATHWAYS_IN_CANCER | 1  1  0.9515  0.8333  0.8333 |
|  | hsa_circ_0002138 | PID_CXCR4_PATHWAY  PID_ERBB1_DOWNSTREAM_PATHWAY  REACTOME_IMMUNE_SYSTEM  KEGG_PATHWAYS_IN_CANCER  KEGG_CALCIUM_SIGNALING_PATHWAY | 1  0.8571  0.8462  0.75  0.75 |
| Module 5 | hsa_circ_0069244 | KEGG_PATHWAYS_IN_CANCER  KEGG_CALCIUM_SIGNALING_PATHWAY  KEGG_PROSTATE_CANCER  KEGG_SMALL_CELL_LUNG_CANCER  KEGG_WNT_SIGNALING_PATHWAY  REACTOME_IMMUNE_SYSTEM | 1  1  1  1  1  0.8846 |
|  | hsa_circ_0017242 | KEGG_CALCIUM_SIGNALING_PATHWAY  KEGG_SMALL_CELL_LUNG_CANCER  KEGG_PATHWAYS_IN_CANCER  KEGG_PROSTATE_CANCER  REACTOME_IMMUNE_SYSTEM  KEGG_GNRH_SIGNALING_PATHWAY  KEGG_WNT_SIGNALING_PATHWAY | 1  1  0.913  0.8889  0.807  0.8  0.7778 |
|  | hsa_circ_0001725 | KEGG_PROSTATE_CANCER  KEGG_GNRH_SIGNALING_PATHWAY  KEGG_SMALL_CELL_LUNG_CANCER  KEGG_CALCIUM_SIGNALING_PATHWAY  KEGG_PATHWAYS_IN_CANCER  REACTOME_IMMUNE_SYSTEM  KEGG_WNT_SIGNALING_PATHWAY | 1  1  1  0.875  0.8696  0.8461  0.7778 |
|  | hsa_circ_0073901 | KEGG_PROSTATE_CANCER  KEGG_SMALL_CELL_LUNG_CANCER  KEGG_PATHWAYS_IN_CANCER  KEGG_CALCIUM_SIGNALING_PATHWAY  KEGG_WNT_SIGNALING_PATHWAY  REACTOME_IMMUNE_SYSTEM  KEGG_GNRH_SIGNALING_PATHWAY | 0.8889  0.8333  0.8261  0.75  0.6667  0.6154  0.6 |
|  | hsa_circ_0007843 | KEGG_CALCIUM_SIGNALING_PATHWAY  KEGG_SMALL_CELL_LUNG_CANCER  KEGG_PATHWAYS_IN_CANCER  KEGG_GNRH_SIGNALING_PATHWAY  KEGG_WNT_SIGNALING_PATHWAY  KEGG_PROSTATE_CANCER  REACTOME_IMMUNE_SYSTEM | 1  1  0.8261  0.8  0.7778  0.7778  0.7692 |
|  | hsa_circ_0086375 | KEGG_SMALL_CELL_LUNG_CANCER  KEGG_GNRH_SIGNALING_PATHWAY  KEGG_PROSTATE_CANCER  KEGG_CALCIUM_SIGNALING_PATHWAY  KEGG_PATHWAYS_IN_CANCER  REACTOME_IMMUNE_SYSTEM  KEGG_WNT_SIGNALING_PATHWAY | 1  0.8  0.7778  0.75  0.7391  0.7308  0.6667 |
|  | hsa_circ_0084143 | KEGG_CALCIUM_SIGNALING_PATHWAY  KEGG_SMALL_CELL_LUNG_CANCER  REACTOME_IMMUNE_SYSTEM  KEGG_GNRH_SIGNALING_PATHWAY  KEGG_PROSTATE_CANCER  KEGG_PATHWAYS_IN_CANCER  KEGG_WNT_SIGNALING_PATHWAY | 0.875  0.8333  0.807  0.8  0.6667  0.5652  0.5556 |
|  | hsa_circ_0008362 | KEGG_SMALL_CELL_LUNG_CANCER  REACTOME_IMMUNE_SYSTEM  KEGG_WNT_SIGNALING_PATHWAY  KEGG_PATHWAYS_IN_CANCER  KEGG_CALCIUM_SIGNALING_PATHWAY | 0.6667  0.5777  0.5556  0.5217  0.5 |
| Module 6 | hsa_circ_0017924 | KEGG_WNT_SIGNALING_PATHWAY | 0.75 |
|  | hsa_circ_0006893 | KEGG_PROSTATE_CANCER | 0.5 |
|  | hsa_circ_0001558 | KEGG_SMALL_CELL_LUNG_CANCER | 0.5 |
| Module 8 | hsa_circ_0001447 | PID_P53_DOWNSTREAM_PATHWAY  KEGG_PATHWAYS_IN_CANCER  KEGG_MAPK_SIGNALING_PATHWAY  PID_NOTCH_PATHWAY  KEGG_SMALL_CELL_LUNG_CANCER | 0.75  0.6  0.5  0.5  0.5 |
|  | hsa_circ_0003759 | PID_P53_DOWNSTREAM_PATHWAY  REACTOME_IMMUNE_SYSTEM  PID_NOTCH_PATHWAY | 0.75  0.7222  0.5 |
|  | hsa_circ_0017924 | KEGG_MAPK_SIGNALING_PATHWAY | 0.5 |
|  | hsa_circ_0001350 | PID_CXCR4_PATHWAY | 0.5 |
| Module 9 | hsa_circ_0037130 | KEGG_CALCIUM_SIGNALING_PATHWAY  PID_P53_DOWNSTREAM_PATHWAY  KEGG_GNRH_SIGNALING_PATHWAY  KEGG_PATHWAYS_IN_CANCER  KEGG_MAPK_SIGNALING_PATHWAY  REACTOME_IMMUNE_SYSTEM  REACTOME_ACTIVATED_TLR4_SIGNALLING  PID_ERBB1_DOWNSTREAM_PATHWAY | 1  1  1  0.9444  0.9444  0.9167  0.9091  0.875 |
|  | hsa_circ_0020399 | KEGG_CALCIUM_SIGNALING_PATHWAY  REACTOME_IMMUNE_SYSTEM  KEGG_MAPK_SIGNALING_PATHWAY  KEGG_PATHWAYS_IN_CANCER  KEGG_GNRH_SIGNALING_PATHWAY  REACTOME_ACTIVATED_TLR4_SIGNALLING  PID_P53_DOWNSTREAM_PATHWAY  PID_ERBB1_DOWNSTREAM_PATHWAY | 0.875  0.7917  0.6667  0.6667  0.6667  0.6364  0.5833  0.5 |
|  | hsa_circ_0004513 | PID_ERBB1_DOWNSTREAM_PATHWAY  PID_P53_DOWNSTREAM_PATHWAY  KEGG_PATHWAYS_IN_CANCER  REACTOME_IMMUNE_SYSTEM  KEGG_CALCIUM_SIGNALING_PATHWAY  KEGG_GNRH_SIGNALING_PATHWAY  KEGG_MAPK_SIGNALING_PATHWAY | 0.75  0.75  0.6667  0.625  0.625  0.5556  0.5 |
|  | hsa_circ_0001119 | KEGG_PATHWAYS_IN_CANCER  PID_P53_DOWNSTREAM_PATHWAY  KEGG_MAPK_SIGNALING_PATHWAY  REACTOME_ACTIVATED_TLR4_SIGNALLING  KEGG_CALCIUM_SIGNALING_PATHWAY  PID_ERBB1_DOWNSTREAM_PATHWAY | 0.6667  0.5833  0.5556  0.5455  0.5  0.5 |
|  | hsa_circ_0007785 | REACTOME_ACTIVATED_TLR4_SIGNALLING  PID_ERBB1_DOWNSTREAM_PATHWAY  KEGG_PATHWAYS_IN_CANCER  REACTOME_IMMUNE_SYSTEM  PID_P53_DOWNSTREAM_PATHWAY  KEGG_MAPK_SIGNALING_PATHWAY | 0.6364  0.625  0.6111  0.5833  0.5833  0.5 |
|  | hsa_circ_0006608 | KEGG_PATHWAYS_IN_CANCER | 0.5 |
|  | hsa_circ_0004539 | KEGG_PATHWAYS_IN_CANCER  KEGG_CALCIUM_SIGNALING_PATHWAY | 0.5  0.5 |
