# Supplementary material for: Systematical Identification of Breast Cancer-Related Circular RNA Modules for Deciphering circRNA Functions Based on the Non-Negative Matrix Factorization Algorithm
Source: Int J Mol Sci. 2019 Feb 20;20(4):919. doi: 10.3390/ijms20040919 (PMC6412941; doi:10.3390/ijms20040919)
Supplement: Supplementary file 1 [file ijms-20-00919-s001.zip › Supplementary Files/Supplementary Table s5.docx]

**Supplementary Table 5. Comparison with MCL approach. A) summary of circRNA modules generated by our approach, B) summary of circRNA modules generated by MCL.**

A

| Modules | All nodes | BC-related nodes  (gene, miRNA and pathway) | BC-related circRNAs | NO. of GO enirchmet |
| --- | --- | --- | --- | --- |
| Modules1 | 222 | 0.05405 | 0.004505 | 169 |
| Modules2 | 415 | 0.02651 | 0 | 380 |
| Modules3 | 172 | 0.04651 | 0.005814 | 148 |
| Modules4 | 233 | 0.03433 | 0 | 128 |
| Modules5 | 382 | 0.03141 | 0 | 150 |
| Modules6 | 141 | 0.08511 | 0 | 61 |
| Modules7 | 171 | 0.03509 | 0 | 16 |
| Modules8 | 216 | 0.04167 | 0 | 124 |
| Modules9 | 331 | 0.0272 | 0 | 280 |

B

| Modules | All nodes | | BC-related nodes  (gene, miRNA and pathway) | | | BC-related circRNAs | | NO. of GO enirchmet |
| --- | --- | --- | --- | --- | --- | --- | --- | --- |
| modules | | All nodes | | BC-related nodes | circRNA | | GO bp | |
| Module1 | | 1678 | | 0.01728 | 0.001192 | | 227 | |
| Module2 | | 60 | | 0 | 0 | | 54 | |
| Module3 | | 40 | | 0 | 0 | | 48 | |
| Module4 | | 36 | | 0 | 0 | | 17 | |
| Module5 | | 25 | | 0 | 0 | | 16 | |
| Module6 | | 22 | | 0 | 0 | | 27 | |
| Module7 | | 20 | | 0 | 0 | | 60 | |
| Module8 | | 20 | | 0.05 | 0 | | 40 | |
