# Supplementary figures and images for: Systematical Identification of Breast Cancer-Related Circular RNA Modules for Deciphering circRNA Functions Based on the Non-Negative Matrix Factorization Algorithm
Source: Int J Mol Sci. 2019 Feb 20;20(4):919. doi: 10.3390/ijms20040919 (PMC6412941; doi:10.3390/ijms20040919)

Module 1

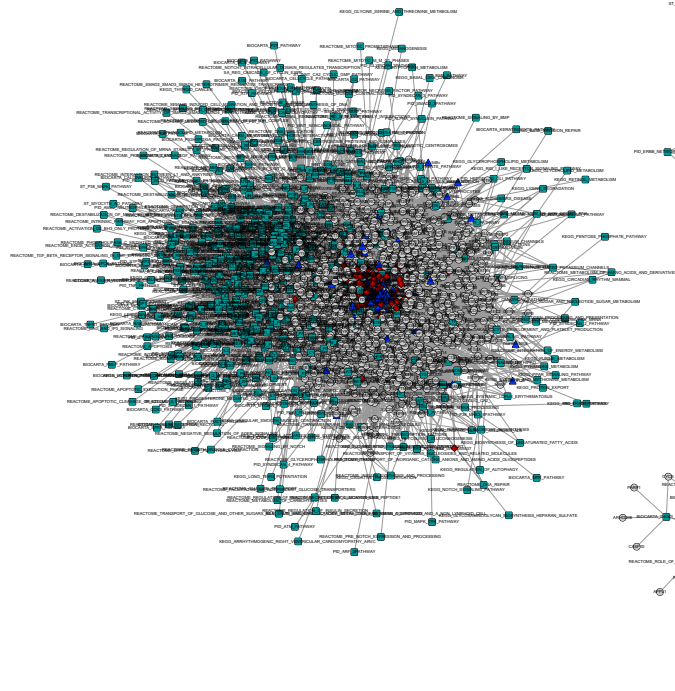

Module 2

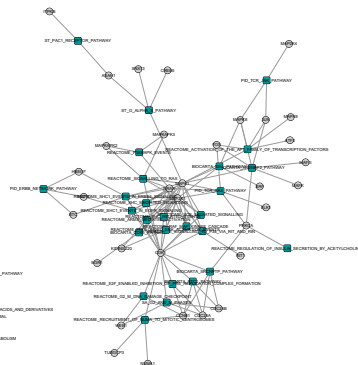

Module 3

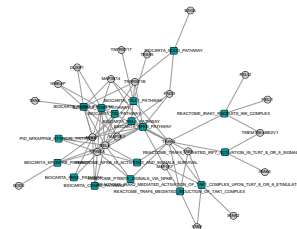

Module 4

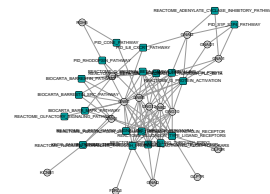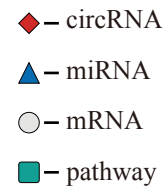

Module 5

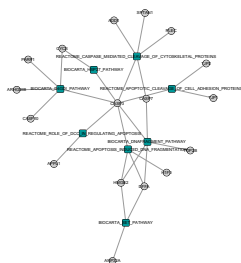

Module 6

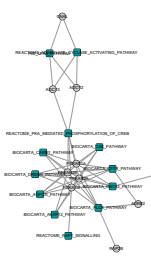

Module 7

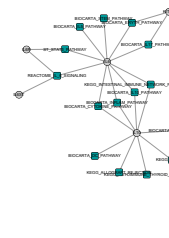

Module 8

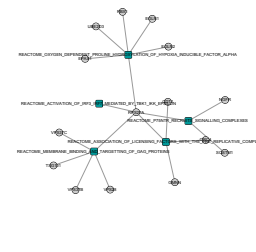

Supplement: Supplementary file 1 [file ijms-20-00919-s001.zip › Supplementary Files/Supplementary Figure s1.pdf]
