# Supplementary material for: Systematical Identification of Breast Cancer-Related Circular RNA Modules for Deciphering circRNA Functions Based on the Non-Negative Matrix Factorization Algorithm
Source: Int J Mol Sci. 2019 Feb 20;20(4):919. doi: 10.3390/ijms20040919 (PMC6412941; doi:10.3390/ijms20040919)
Supplement: Supplementary file 1 [file ijms-20-00919-s001.zip › Supplementary Files/Supplementary Table s2.doc]

**Supplementary Table 2. The relationships between candidate circRNAs and mRNA, miRNAs or pathways.**

| **Modules** | **circRNAs** | **mRNAs** | **miRNAs** | **pathways** |
| --- | --- | --- | --- | --- |
| Module 1 | hsa_circ_0005567 | PTEN | hsa-mir-103a  hsa-mir-200a  hsa-mir-204 | KEGG_PROSTATE_CANCER |
| Module 2 | hsa_circ_0004575 | PIK3CA  PPM1D  SMAD4 | hsa-mir-135a  hsa-mir-185  hsa-mir-190a  hsa-mir-144 | KEGG_PATHWAYS_IN_CANCER  REACTOME_IMMUNE_SYSTEM  KEGG_MAPK_SIGNALING_PATHWAY  KEGG_CALCIUM_SIGNALING_PATHWAY  KEGG_PROSTATE_CANCER  PID_ERBB1_DOWNSTREAM_PATHWAY  KEGG_GNRH_SIGNALING_PATHWAY  PID_CXCR4_PATHWAY |
|  | hsa_circ_0004458 | PIK3CA  PPM1D  SMAD4 | hsa-mir-135a  hsa-mir-185  hsa-mir-190a  hsa-mir-144 | KEGG_PATHWAYS_IN_CANCER  REACTOME_IMMUNE_SYSTEM  KEGG_MAPK_SIGNALING_PATHWAY  KEGG_CALCIUM_SIGNALING_PATHWAY  KEGG_PROSTATE_CANCER  PID_ERBB1_DOWNSTREAM_PATHWAY  KEGG_GNRH_SIGNALING_PATHWAY  PID_CXCR4_PATHWAY |
|  | hsa_circ_0002886 | SMAD4 | hsa-mir-135a  hsa-mir-185  hsa-mir-190a  hsa-mir-144 | KEGG_PATHWAYS_IN_CANCER  REACTOME_IMMUNE_SYSTEM  KEGG_MAPK_SIGNALING_PATHWAY  KEGG_CALCIUM_SIGNALING_PATHWAY  KEGG_PROSTATE_CANCER  PID_ERBB1_DOWNSTREAM_PATHWAY  KEGG_GNRH_SIGNALING_PATHWAY  PID_CXCR4_PATHWAY |
|  | hsa_circ_0007895 | PIK3CA  SMAD4 | hsa-mir-135a  hsa-mir-185  hsa-mir-190a  hsa-mir-144 | KEGG_PATHWAYS_IN_CANCER  REACTOME_IMMUNE_SYSTEM  KEGG_MAPK_SIGNALING_PATHWAY  KEGG_CALCIUM_SIGNALING_PATHWAY  KEGG_PROSTATE_CANCER  PID_ERBB1_DOWNSTREAM_PATHWAY  KEGG_GNRH_SIGNALING_PATHWAY  PID_CXCR4_PATHWAY |
|  | hsa_circ_0027842 | PIK3CA  PPM1D | hsa-mir-135a  hsa-mir-185  hsa-mir-190a  hsa-mir-144 | KEGG_PATHWAYS_IN_CANCER  REACTOME_IMMUNE_SYSTEM  KEGG_MAPK_SIGNALING_PATHWAY  KEGG_CALCIUM_SIGNALING_PATHWAY  KEGG_PROSTATE_CANCER  PID_ERBB1_DOWNSTREAM_PATHWAY  KEGG_GNRH_SIGNALING_PATHWAY  PID_CXCR4_PATHWAY |
|  | hsa_circ_0079753 | PIK3CA | hsa-mir-135a  hsa-mir-185  hsa-mir-190a  hsa-mir-144 | KEGG_PATHWAYS_IN_CANCER  REACTOME_IMMUNE_SYSTEM  KEGG_MAPK_SIGNALING_PATHWAY  KEGG_CALCIUM_SIGNALING_PATHWAY  KEGG_PROSTATE_CANCER  PID_ERBB1_DOWNSTREAM_PATHWAY  KEGG_GNRH_SIGNALING_PATHWAY  PID_CXCR4_PATHWAY |
|  | hsa_circ_0001222 | PIK3CA  PPM1D  SMAD4 | hsa-mir-135a  hsa-mir-185  hsa-mir-190a  hsa-mir-144 | KEGG_PATHWAYS_IN_CANCER  REACTOME_IMMUNE_SYSTEM  KEGG_MAPK_SIGNALING_PATHWAY  KEGG_CALCIUM_SIGNALING_PATHWAY  KEGG_PROSTATE_CANCER  PID_ERBB1_DOWNSTREAM_PATHWAY  KEGG_GNRH_SIGNALING_PATHWAY  PID_CXCR4_PATHWAY |
|  | hsa_circ_0004910 | PIK3CA | hsa-mir-135a  hsa-mir-185  hsa-mir-190a  hsa-mir-144 | KEGG_PATHWAYS_IN_CANCER  REACTOME_IMMUNE_SYSTEM  KEGG_MAPK_SIGNALING_PATHWAY  KEGG_CALCIUM_SIGNALING_PATHWAY  KEGG_PROSTATE_CANCER  PID_ERBB1_DOWNSTREAM_PATHWAY  KEGG_GNRH_SIGNALING_PATHWAY  PID_CXCR4_PATHWAY |
| Module 3 | hsa_circ_0003759 | PPM1D | hsa-mir-135a  hsa-mir-144  hsa-mir-374b  hsa-mir-26a  hsa-mir-7  hsa-mir-224 | KEGG_PATHWAYS_IN_CANCER  PID_P53_DOWNSTREAM_PATHWAY  KEGG_P53_SIGNALING_PATHWAY |
|  | hsa_circ_0001447 | PPM1D | hsa-mir-144  hsa-mir-374b  hsa-mir-26a | KEGG_P53_SIGNALING_PATHWAY |
| Module 4 | hsa_circ_0003614 | AKT1  TSG101 | hsa-mir-378a  hsa-mir-7  hsa-mir-146a | KEGG_PATHWAYS_IN_CANCER  REACTOME_IMMUNE_SYSTEM  PID_ERBB1_DOWNSTREAM_PATHWAY  PID_CXCR4_PATHWAY |
|  | hsa_circ_0007766 | AKT1  TSG101 | hsa-mir-378a  hsa-mir-7  hsa-mir-146a | KEGG_PATHWAYS_IN_CANCER  KEGG_PATHWAYS_IN_CANCER  REACTOME_IMMUNE_SYSTEM  KEGG_CALCIUM_SIGNALING_PATHWAY  PID_ERBB1_DOWNSTREAM_PATHWAY  PID_CXCR4_PATHWAY |
|  | hsa_circ_0002138 | AKT1  TSG101 | hsa-mir-378a  hsa-mir-7  hsa-mir-146a | KEGG_PATHWAYS_IN_CANCER  REACTOME_IMMUNE_SYSTEM  KEGG_CALCIUM_SIGNALING_PATHWAY  PID_ERBB1_DOWNSTREAM_PATHWAY  PID_CXCR4_PATHWAY |
|  | hsa_circ_0003638 | AKT1  TSG101 | hsa-mir-378a  hsa-mir-7  hsa-mir-146a | KEGG_PATHWAYS_IN_CANCER  REACTOME_IMMUNE_SYSTEM  KEGG_CALCIUM_SIGNALING_PATHWAY  PID_ERBB1_DOWNSTREAM_PATHWAY  PID_CXCR4_PATHWAY |
|  | hsa_circ_0003759 | AKT1  TSG101 | hsa-mir-378a  hsa-mir-7  hsa-mir-146a | KEGG_PATHWAYS_IN_CANCER  REACTOME_IMMUNE_SYSTEM  KEGG_CALCIUM_SIGNALING_PATHWAY  PID_ERBB1_DOWNSTREAM_PATHWAY  PID_CXCR4_PATHWAY |
| Module 5 | hsa_circ_0069244 | SMAD4 | hsa-mir-146a  hsa-mir-625  hsa-let-7f  hsa-mir-204 | KEGG_PATHWAYS_IN_CANCER  REACTOME_IMMUNE_SYSTEM  KEGG_CALCIUM_SIGNALING_PATHWAY  KEGG_PROSTATE_CANCER  KEGG_SMALL_CELL_LUNG_CANCER  KEGG_WNT_SIGNALING_PATHWAY |
|  | hsa_circ_0007843 | SMAD4 | hsa-mir-146a  hsa-mir-625  hsa-let-7f  hsa-mir-204 | KEGG_PATHWAYS_IN_CANCER  REACTOME_IMMUNE_SYSTEM  KEGG_CALCIUM_SIGNALING_PATHWAY  KEGG_PROSTATE_CANCER  KEGG_SMALL_CELL_LUNG_CANCER  KEGG_WNT_SIGNALING_PATHWAY  KEGG_GNRH_SIGNALING_PATHWAY |
|  | hsa_circ_0086375 | SMAD4 | hsa-mir-146a  hsa-mir-625  hsa-let-7f  hsa-mir-204 | KEGG_PATHWAYS_IN_CANCER  REACTOME_IMMUNE_SYSTEM  KEGG_CALCIUM_SIGNALING_PATHWAY  KEGG_PROSTATE_CANCER  KEGG_SMALL_CELL_LUNG_CANCER  KEGG_WNT_SIGNALING_PATHWAY  KEGG_GNRH_SIGNALING_PATHWAY |
|  | hsa_circ_0073901 | SMAD4 | hsa-mir-146a  hsa-mir-625  hsa-let-7f  hsa-mir-204 | KEGG_PATHWAYS_IN_CANCER  REACTOME_IMMUNE_SYSTEM  KEGG_CALCIUM_SIGNALING_PATHWAY  KEGG_SMALL_CELL_LUNG_CANCER  KEGG_WNT_SIGNALING_PATHWAY |
|  | hsa_circ_0008362 | SMAD4 | hsa-mir-146a  hsa-mir-625  hsa-let-7f | KEGG_PATHWAYS_IN_CANCER  REACTOME_IMMUNE_SYSTEM  KEGG_CALCIUM_SIGNALING_PATHWAY  KEGG_SMALL_CELL_LUNG_CANCER  KEGG_WNT_SIGNALING_PATHWAY |
|  | hsa_circ_0001725 | SMAD4 | hsa-mir-146a  hsa-mir-625  hsa-let-7f  hsa-mir-204 | KEGG_PATHWAYS_IN_CANCER  REACTOME_IMMUNE_SYSTEM  KEGG_CALCIUM_SIGNALING_PATHWAY  KEGG_PROSTATE_CANCER  KEGG_WNT_SIGNALING_PATHWAY  KEGG_GNRH_SIGNALING_PATHWAY |
|  | hsa_circ_0017242 | null | hsa-mir-146a  hsa-mir-625  hsa-let-7f  hsa-mir-204 | KEGG_PATHWAYS_IN_CANCER  REACTOME_IMMUNE_SYSTEM  KEGG_CALCIUM_SIGNALING_PATHWAY  KEGG_PROSTATE_CANCER  KEGG_GNRH_SIGNALING_PATHWAY  KEGG_SMALL_CELL_LUNG_CANCER  KEGG_WNT_SIGNALING_PATHWAY |
| Module 6 | hsa_circ_0044177 | SMAD4 | hsa-mir-449a  hsa-mir-182 | KEGG_WNT_SIGNALING_PATHWAY |
|  | hsa_circ_0069492 | SMAD4 | hsa-mir-449a  hsa-mir-182  hsa-mir-34b | null |
|  | hsa_circ_0001558 | null | hsa-mir-449a  hsa-mir-182 | KEGG_SMALL_CELL_LUNG_CANCER  KEGG_WNT_SIGNALING_PATHWAY |
|  | hsa_circ_0017924 | SMAD4 | hsa-mir-449a  hsa-mir-182 | KEGG_WNT_SIGNALING_PATHWAY |
| Module 8 | hsa_circ_0001447 | ERBB2 | null | KEGG_PATHWAYS_IN_CANCER  PID_P53_DOWNSTREAM_PATHWAY  KEGG_SMALL_CELL_LUNG_CANCER  PID_NOTCH_PATHWAY |
| Module 9 | hsa_circ_0037130 | null | hsa-mir-216b  hsa-mir-374b  hsa-mir-7  hsa-mir-374b  hsa-mir-185 | KEGG_PATHWAYS_IN_CANCER  REACTOME_IMMUNE_SYSTEM  KEGG_MAPK_SIGNALING_PATHWAY  KEGG_CALCIUM_SIGNALING_PATHWAY  PID_ERBB1_DOWNSTREAM_PATHWAY  PID_P53_DOWNSTREAM_PATHWAY  KEGG_GNRH_SIGNALING_PATHWAY  REACTOME_ACTIVATED_TLR4_SIGNALLING |
|  | hsa_circ_0020399 | null | hsa-mir-216b  hsa-mir-374b  hsa-mir-7  hsa-mir-374b  hsa-mir-185 | KEGG_PATHWAYS_IN_CANCER  REACTOME_IMMUNE_SYSTEM  KEGG_MAPK_SIGNALING_PATHWAY  KEGG_CALCIUM_SIGNALING_PATHWAY  PID_ERBB1_DOWNSTREAM_PATHWAY  PID_P53_DOWNSTREAM_PATHWAY  KEGG_GNRH_SIGNALING_PATHWAY  REACTOME_ACTIVATED_TLR4_SIGNALLING |
|  | hsa_circ_0007785 | null | hsa-mir-216b  hsa-mir-374b  hsa-mir-7  hsa-mir-374b  hsa-mir-185 | KEGG_PATHWAYS_IN_CANCER  REACTOME_IMMUNE_SYSTEM  KEGG_MAPK_SIGNALING_PATHWAY  PID_ERBB1_DOWNSTREAM_PATHWAY  PID_P53_DOWNSTREAM_PATHWAY  REACTOME_ACTIVATED_TLR4_SIGNALLING |
|  | hsa_circ_0004513 | null | hsa-mir-216b  hsa-mir-374b  hsa-mir-7  hsa-mir-374b  hsa-mir-185 | KEGG_PATHWAYS_IN_CANCER  REACTOME_IMMUNE_SYSTEM  KEGG_MAPK_SIGNALING_PATHWAY  KEGG_CALCIUM_SIGNALING_PATHWAY  PID_ERBB1_DOWNSTREAM_PATHWAY  PID_P53_DOWNSTREAM_PATHWAY  KEGG_GNRH_SIGNALING_PATHWAY |
|  | hsa_circ_0001119 | null | hsa-mir-216b  hsa-mir-374b  hsa-mir-7  hsa-mir-374b  hsa-mir-185 | KEGG_PATHWAYS_IN_CANCER  KEGG_MAPK_SIGNALING_PATHWAY  KEGG_CALCIUM_SIGNALING_PATHWAY  PID_ERBB1_DOWNSTREAM_PATHWAY  PID_P53_DOWNSTREAM_PATHWAY  REACTOME_ACTIVATED_TLR4_SIGNALLING |
